# Kinetic Characterization and Phosphoregulation of the *Francisella tularensis* 1-Deoxy-D-Xylulose 5-Phosphate Reductoisomerase (MEP Synthase)

**DOI:** 10.1371/journal.pone.0008288

**Published:** 2009-12-14

**Authors:** Safdar Jawaid, Heather Seidle, Weidong Zhou, Hafsa Abdirahman, Maher Abadeer, Joseph H. Hix, Monique L. van Hoek, Robin D. Couch

**Affiliations:** 1 Department of Chemistry and Biochemistry, George Mason University, Manassas, Virginia, United States of America; 2 Department of Molecular and Microbiology, George Mason University, Manassas, Virginia, United States of America; 3 National Center for Biodefense and Infectious Diseases, George Mason University, Manassas, Virginia, United States of America; 4 Center for Applied Proteomics and Molecular Medicine, George Mason University, Manassas, Virginia, United States of America; University of Canterbury, New Zealand

## Abstract

Deliberate and natural outbreaks of infectious disease underscore the necessity of effective vaccines and antimicrobial/antiviral therapeutics. The prevalence of antibiotic resistant strains and the ease by which antibiotic resistant bacteria can be intentionally engineered further highlights the need for continued development of novel antibiotics against new bacterial targets. Isoprenes are a class of molecules fundamentally involved in a variety of crucial biological functions. Mammalian cells utilize the mevalonic acid pathway for isoprene biosynthesis, whereas many bacteria utilize the methylerythritol phosphate (MEP) pathway, making the latter an attractive target for antibiotic development. In this report we describe the cloning and characterization of *Francisella tularensis* MEP synthase, a MEP pathway enzyme and potential target for antibiotic development. *In vitro* growth-inhibition assays using fosmidomycin, an inhibitor of MEP synthase, illustrates the effectiveness of MEP pathway inhibition with *F. tularensis*. To facilitate drug development, *F. tularensis* MEP synthase was cloned, expressed, purified, and characterized. Enzyme assays produced apparent kinetic constants (*K_M_^DXP^* = 104 µM, *K_M_^NADPH^* = 13 µM, *k_cat_^DXP^* = 2 s^−1^, *k_cat_^NADPH^* = 1.3 s^−1^), an IC_50_ for fosmidomycin of 247 nM, and a *K_i_* for fosmidomycin of 99 nM. The enzyme exhibits a preference for Mg^+2^ as a divalent cation. Titanium dioxide chromatography-tandem mass spectrometry identified Ser177 as a site of phosphorylation. S177D and S177E site-directed mutants are inactive, suggesting a mechanism for post-translational control of metabolic flux through the *F. tularensis* MEP pathway. Overall, our study suggests that MEP synthase is an excellent target for the development of novel antibiotics against *F. tularensis*.

## Introduction

The US Centers for Disease Control and Prevention (CDC) classify biothreat agents based upon their ease of dissemination, associated morbidity/mortality rates, projected social impact, and emergency response procedures. Category A agents (i.e. those of highest concern) include *Bacillus anthracis* (the causative agent of anthrax), *Yersinia pestis* (plague), and *Francisella tularensis* (tularemia), while category B agents (exhibiting lower morbidity/mortality rates) include *Brucella* species (brucellosis), *Burkholderia mallei* (glanders), and *Burkholderia pseudomallei* (melioidosis). The 1984 Rajneeshee *Salmonella* attack, 2001 anthrax letter attacks, 2003 SARS outbreak, and 2009 H1N1 swine flu pandemic illustrate our vulnerability to both deliberate and natural outbreaks of infectious disease and underscore the necessity of effective vaccines and antimicrobial/antiviral therapeutics. The prevalence of antibiotic resistant strains and the ease by which antibiotic resistance can be engineered into bacteria further highlights the need for continued development of novel antibiotics against new bacterial targets.

**Figure 1:**
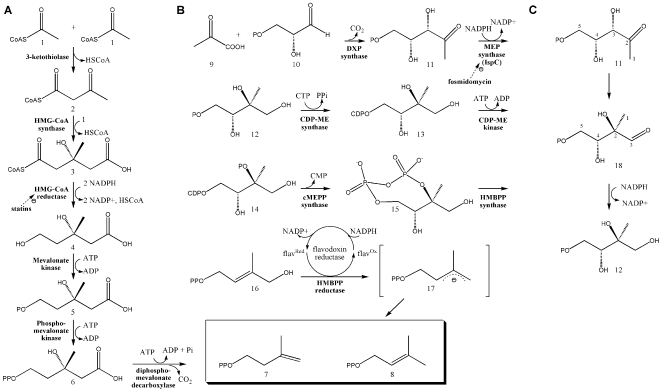
The two isoprene biosynthetic pathways. A) The MVA Pathway. The first two enzymes of the MVA pathway condense 3 molecules of acetyl-CoA (1) to form 3-hydroxy-3-methylglutaryl CoA (HMG-CoA) (3), which is subsequently reduced to MVA (4) by HMG-CoA reductase [20], [21]. MVA is phosphorylated twice then decarboxylated to yield IPP (7) [22]–[24], which is converted to DMAPP (8) by an isomerase [25]. B) The MEP Pathway. Condensation of pyruvate (9) with glyceraldehyde 3-phosphate (10) yields 1-deoxy-D-xylulose 5-phosphate (DXP; (11)) [26], an intermediate with a role in *E. coli* vitamin B1 and B6 biosynthesis [27]–[30] and isoprene biosynthesis. 1-Deoxy-D-xylulose 5-phosphate reductoisomerase (also called MEP synthase or IspC) catalyzes the reduction and rearrangement of 11 to yield MEP (12) [5], the first committed step in the *E. coli* MEP pathway. The next enzyme, CDP-ME synthase, converts MEP into 4-(cytidine 5′-diphospho)-2-C-methyl-D-erythritol (CDP-ME; (13)). CDP-ME kinase then phosphorylates CDP-ME, which is subsequently cyclized (coupled with the loss of CMP) by cMEPP synthase to yield 2-C-methyl-D-erythritol 2,4-cyclodiphosphate (15) [31]–[35]. A reductive ring opening of 15 produces 1-hydroxy-2-methyl-2-butenyl diphosphate (HMBPP; (16)) [12], [36]–[39], which is subsequently reduced to both IPP and DMAP in a ∼5:1 ratio [2], [40]–[45]. C) The reaction catalyzed by MEP synthase. Isomerization via cleavage of the bond between C3 and C4 and formation of a new bond between C2 and C4 produces the intermediate 2-C-methyl-D-erythrose 4-phosphate (18) [46], [47], which is subsequently reduced to yield MEP (12).

Isoprenoids are a class of molecules fundamentally involved in a variety of crucial biological functions including electron transport (quinones), cell wall biosynthesis (dolichols), signal transduction (prenylated proteins), and the regulation of membrane fluidity (hopanoids and cholesterol). Despite their structural and functional diversity, all isoprenoids are derived from two building blocks, isopentenyl diphosphate (IPP) and dimethylallyl diphosphate (DMAPP), which originate from either the mevalonic acid (MVA) or methylerythritol phosphate (MEP) pathway (Fig. 1). Because mammalian cells exclusively utilize the MVA pathway, enzymes within the MEP pathway make an attractive target for the development of novel antimicrobials (reviewed in [1]–[3]).

Genome sequences reveal that *Francisella*, *Brucella*, *Bacillus*, *Burkholderia*, and *Yersinia* each harbor MEP pathway genes, but little else is known about isoprene biosynthesis in these biothreat agents. In this report we describe the characterization of *F. tularensis* MEP synthase, a MEP pathway enzyme and potential target for drug development. The *F. tularensis* MEP synthase gene was cloned, expressed in *Escherichia coli*, and the recombinant protein was purified and enzymatically characterized. Post-translational modification (phosphorylation) was revealed by protein mass spectrometry and correlated with the loss of enzyme activity, suggesting a possible regulatory mechanism. The stability of *F. tularensis* MEP synthase in high-throughput type assays amenable to drug development was also evaluated. Overall, our results suggest that MEP synthase is an excellent target for the development of novel antibiotics against *F. tularensis*.

## Materials and Methods

### Bacterial strains and growth conditions

The following reagents were obtained through the NIH Biodefense and Emerging Infections Research Resources Repository, NIAID, NIH: *Francisella tularensis* subsp. *holarctica* Strain LVS (FSC 155) and *Francisella tularensis* subsp. *novicida* Strain Utah 112. Each was cultured at 37°C in tryptic soy broth supplemented with 0.1% cysteine or in modified Chamberlain's Defined Medium (0.4 g/L L-Arginine, 0.4 g/L L-Aspartic acid, 0.2 g/L L-Cysteine, 0.2 g/L L-Histidine, 0.4 g/L L-Isoleucine, 0.4 g/L L-Leucine, 0.4 g/L L-Lysine, 0.4 g/L L-Methionine, 2.0 g/L L-Proline, 0.4 g/L L-Serine, 2.0 g/L L-Threonine, 0.4 g/L L-Tyrosine, 0.4 g/L L-Valine, 0.04 g/L Spermine phosphate, 0.004 g/L Thiamine HCl, 0.002 g/L L-Calcium pantothenate, 4.0 g/L Glucose, 10.0 g/L NaCl, 0.135 g/L MgSO_4_ 7H_2_O, 1.0 g/L KH_2_PO_4_, 1.0 g/L K_2_HPO_4_, 1.92 g/L sodium citrate, 0.02 g/L FeSO_4_ 7H_2_O, pH 6.2). *Escherichia coli* BL21 CodonPlus (DE3)-RIL cells (Stratagene, La Jolla, CA) were used for recombinant protein expression whereas *E. coli* TOP10 (Invitrogen, Carlsbad, CA) or XL1Blue (Stratagene) cells were used for general cloning procedures. *E. coli* was grown at 37°C in Luria-Bertani (LB) media with constant shaking (250 rpm). Protein expression was performed in LB media containing ampicillin (100 µg/ml) and chloramphenicol (50 µg/ml). Solid media were prepared by addition of 1.5% (wt/vol) agar.

### Fosmidomycin IC_50_ determination

The half-maximal inhibitory concentration (IC_50_) was determined via a dose-response plot of fractional growth (OD_595_ in the presence of inhibitor/OD_595_ in the absence of the inhibitor) as a function of fosmidomycin concentration. An overnight culture of *F. tularensis* subsp. *novicida* was harvested by centrifugation, diluted to an OD_595_ = 1.0, then used to inoculate modified Chamberlain's Defined Medium (using a 96-well plate; 17 µL of inoculum was added to 158 µL media containing varying concentrations of fosmidomycin). All conditions were evaluated in duplicate. Bacterial growth (OD_595_ at 37°C) was determined using a Beckman Coulter DTX800 plate reader. A nonlinear regression analysis was carried out on the data obtained using GraphPad PRISM version 4.00 for Windows (GraphPad Software Inc., San Diego, CA) and the equation F  = 1/(1+[I]/IC_50_) where F  =  fractional growth and [I]  =  inhibitor concentration.

### Genomic and Plasmid DNA isolation

*F. tularensis* subsp. *holarctica* genomic DNA was isolated using a Wizard Genomic DNA Purification Kit (Promega, Madison, WI), per the manufacturer's instructions. Plasmid DNA was isolated from *E. coli* using a GenElute Plasmid miniprep kit (Sigma-Aldrich, St. Louis, MO).

### Construction of the *F. tularensis* MEP synthase expression plasmid

The MEP synthase coding region (*ispC*) was identified in the *F. tularensis* subsp. *holarctica* LVS genome (accession number NC_007880) via a BLAST search using the *E. coli* K12 homologous sequence as the query. Polymerase chain reaction (PCR) primer pairs, designed to flank *ispC* (FtIspC-f; 5′- CACCATGTTTAAAAAAACTAAGATTAC -3′ and FtIspC-r; 5′- CCCCAAAACAGAATCCACATATTC -3′), were purchased from Sigma-Genosys (The Woodlands, TX) and used to amplify the gene from *F. tularensis* subsp. *holarctica* LVS genomic DNA. FtIspC-f contains four additional 5′ residues (CACC) to facilitate the unidirectional insertion of the PCR product into plasmid pET101/D-TOPO (Invitrogen). FtIspC-r is designed to eliminate the stop codon in the PCR product to permit the expression of a C-terminal His-tagged MEP synthase protein. PCR was performed with Platinum Pfx polymerase (Invitrogen) and the following parameters: 2 minutes at 94°C followed by 22 cycles of 15 sec at 94°C, 30 sec at 54°C, 1.5 min at 68°C, and a final elongation of 10 min at 68°C. The PCR product was purified using the Qiaquick PCR Purification Kit (Qiagen, Valencia, CA) and cloned into pET101/D-TOPO to create pFtIspC. Restriction mapping and DNA sequencing were used to confirm the fidelity of the PCR and the correct construction of the plasmid. pFtIspC was transformed into chemically competent *E. coli* BL21 CodonPlus (DE3)-RIL cells to express the protein.

### Expression and purification of *F. tularensis* MEP synthase

A one liter shake flask was used for protein expression (37°C, 250 rpm). The flask was inoculated with a 10 mL overnight culture of *E. coli* BL21 CodonPlus (DE3)-RIL containing pFtIspC and upon reaching an OD_600_ of 1.1, protein expression was induced by the addition of 0.5 mM isopropyl β-D-thiogalactopyranoside (IPTG). After incubation for an additional 18 hours, cells were harvested by centrifugation and stored at −80°C. To purify the His-tagged protein, the cell pellet was thawed then cells were lysed using Lysis Buffer A (100 mM Tris pH 8, .032% lysozyme; 3 mL per mg cell pellet), followed by Lysis Buffer B (0.1 M CaCl_2_, 0.1 M MgCl_2_, 0.1 M NaCl, .020% DNase; 0.3 mL per mg cell pellet). Clarified cell lysate was obtained by centrifugation (48,000 x g, 20 min) then passed through a TALON immobilized metal affinity chromatography column (Clontech Laboratories, Mountain View, CA). The column was washed with 15 column volumes of 1X equilibration buffer (50 mM HEPES pH 7.5, 300 mM NaCl), 10 column volumes of 1X wash buffer (50 mM HEPES pH 7.5, 300 mM NaCl, 10 mM imidazole), 10 column volumes of 2X wash buffer (100 mM HEPES pH 7.5, 600 mM NaCl, 20 mM imidazole), and the His-tagged protein was then eluted with 5 column volumes of 1X elution buffer (150 mM imidazole pH 7.0, 300 mM NaCl). Buffer was exchanged by addition of 0.1 M Tris pH 7.5, 1 mM NaCl, 5 mM DTT while concentrating the protein by ultrafiltration. Protein concentration was determined using the Advanced Protein Assay Reagent (Cytoskeleton, Denver, CO) with γ-globulins (Sigma-Aldrich) as the standard. The protein was visualized via Coomassie stained SDS-PAGE and a Western blot with an anti-His antibody (Qiagen). The yield of purified MEP synthase averaged 5–10 mg per 1 L LB shake flask.

### Size-Exclusion Chromatography

The molecular mass of MEP synthase and the mutant derivatives were estimated by loading 1 mg of protein onto a Sephacryl 200HR (Sigma Aldrich, St. Louis, MO) size-exclusion chromatography column equilibrated with 0.1 M Tris pH 7.5, 1 mM NaCl, 5 mM DTT (flow rate of 2 mL/min) and calibrated with a gel filtration standard kit purchased from Bio-Rad (Hercules, CA). Blue dextran was used to determine the void volume of the column.

### Mutagenesis

The Ser_177_ to Asp_177_ mutant of MEP synthase was created via PCR based site directed mutagenesis using primers FT-IspC-SD177-FP (5′ TTA ACA GCA gaT GGA GGT CCT TTT AG 3′; lowercase residues indicate site of mutation) and FT-IspC-SD177-RP (5′ AGG ACC TCC Atc TGC TGT TAA AAT TAT C 3′), whereas the Ser_177_ to Glu_177_ mutant was created using primer pairs FT-IspC-SE177-FP (5′ TTA ACA GCA gaa GGA GGT CCT TTT AGA G 3′) and FT-IspC-SE177-RP (5′ AGG ACC TCC ttc TGC TGT TAA AAT TAT C 3′). Each primer pair is oriented outward from a central overlapping region. In the first step of the mutagenesis, four unidirectional PCRs were performed with each of the four individual PCR primers (FT-IspC-SD177-FP, FT-IspC-SD177-RP, FT-IspC-SE177-FP, or FT-IspC-SE177-RP) using the following parameters: 5 minutes at 95°C followed by 30 cycles of 30 sec at 95°C, 30 sec at 60°C, 40 sec at 72°C, and a final elongation of 7 min at 72°C. In the second step, 5 µL of each PCR product was appropriately combined with the product obtained from the companion primer (i.e. the product using FT-IspC-SD177-FP was combined with the FT-IspC-SD177-RP product, whereas the products obtained from the PCRs with FT-IspC-SE177-FP and FT-IspC-SE177-RP were combined) and each mixture served as template for a subsequent PCR using T7 forward and reverse primers. PCR conditions were as follows: 5 minutes at 95°C followed by 5 cycles of 30 sec at 95°C, 30 sec at 60°C, 40 sec at 72°C, then 25 cycles of 30 sec at 95°C, 30 sec at 52°C, 40 sec at 72°C and a final elongation of 10 min at 72°C. In the third step, the PCR products from step 2 were amplified using primers FtIspC-f and FtIspC-r (described above), and the resulting products were cloned into pET101/D-TOPO, transformed into XL1Blue cells, and sequence confirmed by restriction digestion and nucleotide sequencing. Each expression vector was introduced into chemically competent *E. coli* BL21 CodonPlus (DE3)-RIL cells to express the C-terminal His-tagged protein. The S177D and S177E mutants were purified as described for the wildtype His-tagged MEP synthase.

### Fluorescence spectroscopy

Fluorescence spectra of MEP synthase and the mutant derivatives were measured using a Fluoromax-3 fluorometer (Horiba Jobin Yvon) at an excitation wavelength of 290 nm using cuvettes with an optical path length of 1 cm. The emission spectra of protein samples with a concentration of 5 µM in 0.1 M Tris pH 7.5, 1 mM NaCl, 5 mM DTT were measured from 310 to 400 nm (excitation and emission slit width  = 5 nm). The temperature was maintained at 30°C. All fluorescence spectra were corrected for background with pure buffer.

### Enzyme Assays

MEP synthase activity was evaluated at 22°C by spectrophotometrically monitoring the enzyme catalyzed oxidation of NADPH using an assay derived from that described by Takahashi *et al* [4]. All assays were performed in triplicate. To determine the apparent K_M_ for 1-deoxy-D-xylulose 5-phosphate (DXP), assay mixtures (200 µL) contained 100 mM Tris pH 7.8, 25 mM MgCl_2_, 0.15 mM NADPH, 7 µM MEP synthase, and a variable concentration of DXP (Echelon Biosciences, Salt Lake City, UT). To determine cation specificity, assays were performed with 25 mM MgCl_2_, CaCl_2_, CoCl_2_, CuCl_2_, MnCl_2_, ZnCl_2_, or NiCl_2_. To determine the apparent *K_M_* for NADPH, assays were performed with fixed DXP concentration (0.4 mM) and variable NADPH concentration. Nonlinear regression fitting to the Michaelis-Menten equation was used to determine the kinetic constants. Half-maximal inhibition (IC_50_) by fosmidomycin was determined using a plot of enzyme fractional activity as a function of inhibitor concentration. A plot of *K_M_^app^* as a function of inhibitor concentration was used to determine the *K_i_* for fosmidomycin (negative value of the x intercept). Because fosmidomycin is a slow, tight binding inhibitor [5], the enzyme was preincubated with fosmidomycin for 10 minutes prior to addition of substrate. High-throughput assays were performed using 96-well plates with assay volumes adjusted to 100 µL. The Z-factor was calculated as described by Zhang *et al* [6] with fosmidomycin as the inhibitor control.

### Mass spectrometry method for phosphopeptide identification

To obtain MEP synthase for phosphopeptide analysis, protein expression and purification was essentially as described above, with the exception of using 0.01 mM IPTG for induction. Purified MEP synthase was reduced with 10 mM dithiothreitol (DTT), alkylated by iodoacetamide (50 mM), and then digested by trypsin (Promega) in buffer containing ammonium bicarbonate (50 mM, pH 9) and urea (2 M). The digestion mixture was then desalted by a SepPak column (Waters, Milford, MA). Phosphopeptides were enriched from the tryptic peptides by a TiO_2_ column as described by Thingholm *et al* [7] with modification. In brief, 30 cm fused silica capillary tubing (360 µm OD, 200 µm ID, Polymicro Technologies, Phoenix, AZ) was attached to the frit end of Inline MicroFilter Assembly (Upchurch Scientific), and TiO_2_ loose media (GL Sciences, Inc) was slurry-packed into the tubing using a Pressure Cell (Brechbühler Inc.) to form a 200 µm ×2 cm TiO_2_ column. The SepPak-cleaned sample was mixed with equal volume of Loading Buffer (200 mg/mL 2,5-dihydroxybenzoic acid (DHB), 5% trifluoroacetic acid (TFA), 80% acetonitrile), and loaded into TiO_2_ column by Pressure Cell with flow rate of 3 µL per minute. The column was washed with 200 µL Wash Buffer 1 (40 mg/ml DHB, 2% TFA, 80% acetonitrile) and 2×200 µL Wash Buffer 2 (2% TFA, 50% acetonitrile) to remove non-phosphopeptides. Phosphopeptides were then eluted off the column by Elution Buffer (5% ammonia solution). Ammonia in the eluate was evaporated by SpeedVac (∼3 min), acidified by adding glacial acetic acid to a final concentration of 2%, and desalted using a ZipTip (Millipore). The purified phosphopeptides were analyzed by reversed-phase liquid chromatography nanospray tandem mass spectrometry (LC-MS/MS) using an LTQ-Orbitrap mass spectrometer (ThermoFisher) using previously described methods [8]. Tandem mass spectra were searched using the program SEQUEST (Bioworks software, Thermo) with full tryptic cleavage constraints, static cysteine alkylation by iodoacetamide, and variable phosphorylation of Ser/Thr/Tyr. Phosphopeptide identification was determined using database match scoring criteria filters followed by manual evaluation of the raw data, as described [8].

### Molecular Modeling

*F. tularensis* subsp. *holarctica* LVS MEP synthase (protein accession number CAJ78974) was homology-modeled using SWISS-MODEL [9] (http://swissmodel.expasy.org/) in automated modeling mode. The template used for modeling was identified via the template identification tool (with default parameters), which performs a primary sequence comparison of the query sequence with those in a structural database. The resolved structure that exhibited the greatest sequence homology with *F. tularensis* MEP synthase was chosen as the template for modeling (*E. coli* MEP synthase; PDB ID# 1T1R, BLAST e value = 3×10^−94^, 48% identity and 66% homology with the *F. tularensis* sequence). The quality of the *F. tularensis* MEP synthase model was evaluated using ProQRes[10], which uses atom-atom contacts, residue-residue contacts, solvent accessibility, and secondary structure information to assign an accuracy score from 0 (unreliable) to 1 (reliable). Swiss-PdbViewer 4.0 (http://spdbv.vital-it.ch/) was used to visualize and annotate the model.

## Results and Discussion

### The *F. tularensis* MEP pathway as an antimicrobial target

**Figure 2:**
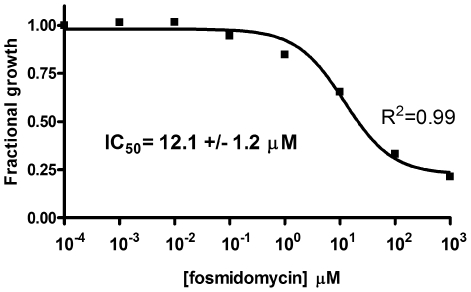
Dose-response plot of *F. tularensis* subsp. *novicida* growth as a function of fosmidomycin concentration. Fractional growth was calculated as the ratio of cell density (OD_595_) in the presence of inhibitor to cell density in the absence of inhibitor. Nonlinear regression fitting indicates half maximal activity at 12 µM. The goodness-of-fit (R^2^) value is indicated. Growth curves are presented in supportive Fig. S1.

Since MEP pathway orthologs are not found in the human genome, the pathway makes an attractive target for the development of novel antibiotics. To assess if MEP pathway inhibition would restrict the *in vitro* proliferation of *F. tularensis* subsp. *novicida*, we monitored bacterial growth in media supplemented with fosmidomycin[11], a strong and specific inhibitor of MEP synthase (*E. coli* genetically engineered to use mevalonate for IPP biosynthesis (Fig. 1) is unaffected by fosmidomycin when the culture medium is supplemented with mevalonate, but growth is inhibited by fosmidomycin when mevalonate is excluded[12], illustrating the specificity of fosmidomycin for the MEP pathway. Dose-dependent inhibition of purified *E. coli* MEP synthase[11] and a resolved crystal structure of *E. coli* MEP synthase bound to the inhibitor[13] further illustrate this specificity). As illustrated in the dose-response plot shown in Fig. 2, fosmidomycin inhibits *F. tularensis* growth, with half-maximal inhibition at 12 µM (2.2 µg/mL). A transposon mutant library of *F. tularensis* [14] further confirms the essentiality of the pathway, as MEP pathway knockouts are lethal, further validating the pathway as an attractive target in *F. tularensis*. To facilitate drug development, we next set out to clone and evaluate MEP synthase.

### Cloning, expression, and purification of *F. tularensis* MEP synthase

**Figure 3:**
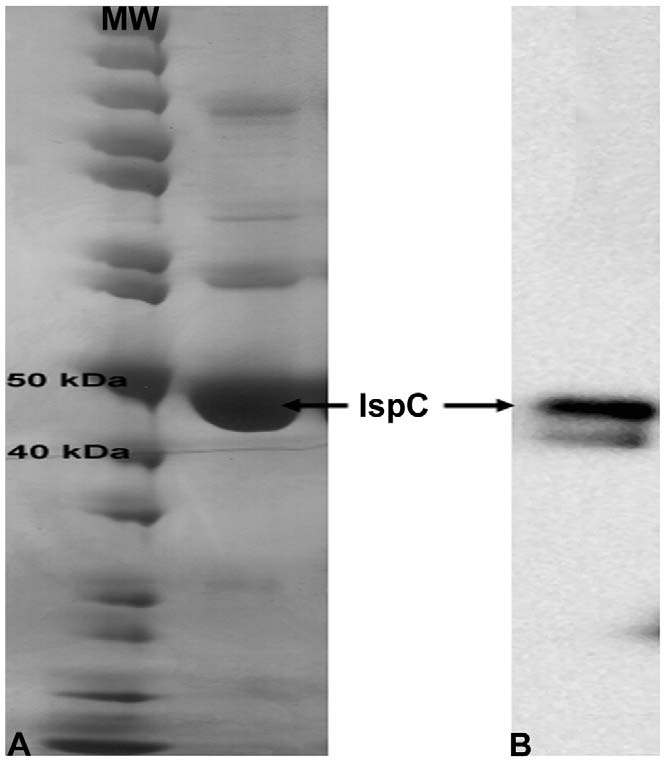
Purification of recombinant *F. tularensis* MEP synthase. A) Coomassie stained SDS-PAGE showing a molecular weight marker (MW) and purified His-tagged MEP synthase. His-tagged MEP synthase has a predicted molecular weight of 46.4 kDa. B) Western blot hybridization analysis of purified MEP synthase using an anti-His antibody results in an intense band of the expected size. The appearance of a weak, smaller molecular weight band suggests that some degradation may have occurred.

The *F. tularensis* subsp. *holarctica* LVS *ispC* gene, identified via a BLAST search of the genome using the *E. coli* K12 homologous sequence, is 1158 bp in length and encodes a polypeptide of 385 amino acids with a calculated molecular mass of 42.7 kDa. The subsp. *holarctica* MEP synthase amino acid sequence shares 99.7, 99.0, and 99.7% identity with the MEP synthase sequence from subsp. *tularensis*, subsp. *novicida*, and subsp. *mediasiatica*, respectively. PCR primer pairs, designed to flank the subsp. *holarctica ispC*, were used to amplify the gene. The PCR product was cloned into an expression plasmid engineered to express a C-terminal His-tagged protein in *E. coli*. Purified protein was visualized by SDS-PAGE and Western blot hybridization using an anti-His antibody (Fig. 3). Size-exclusion chromatography using a calibrated column revealed that *F. tularensis* MEP synthase exists in solution as a dimer of ∼94 kDa (Fig. S2), similar to MEP synthase from *Mycobacterium tuberculosis* [15] and *Synechocystis* sp. PCC6803[16].

### Kinetic characterization of *F. tularensis* MEP synthase

**Figure 4:**
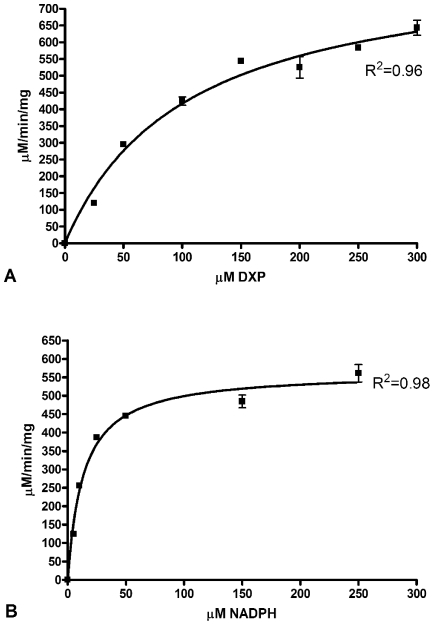
The substrate dependent activity of *F. tularensis* MEP synthase. Michaelis-Menten plots of reaction velocity as a function of A) DXP concentration and B) NADPH concentration were used to derive the kinetic parameters listed in Table 1. The solid line represents the nonlinear least-squares best fit of the data to the Michaelis-Menten equation. The R^2^ value for each plot is indicated.

**Figure 5:**
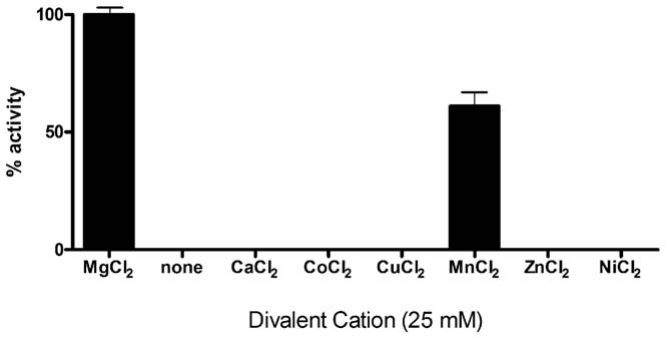
The divalent cation specificity of *F. tularensis* MEP synthase. Enzyme assays were performed with several different divalent cations at a fixed DXP (150 µM) and NADPH (100 µM) concentration. Relative enzyme activity reveals the preference of the enzyme for Mg^+2^.

The kinetic activity of purified MEP synthase was spectrophotometrically evaluated by monitoring the substrate dependent enzyme catalyzed oxidation of NADPH (Fig. 1C). Nonlinear regression fitting of enzyme velocity versus substrate concentration was used to determine the apparent kinetic constants (Fig. 4 and Table 1). The *K_M_^app^* for 1-deoxy-D-xylulose 5-phosphate (DXP) was obtained using assays performed with a saturating concentration of NADPH (150 µM), whereas the *K_M_^app^* for NADPH was determined using assays with saturating levels of DXP (400 µM). The *K_M_^app, DXP^* and *K_M_^app, NADPH^* for recombinant *F. tularensis* MEP synthase are consistent with values reported for the enzyme from *E. coli*, *M. tuberculosis*, and *Synechocystis* sp. PCC6803 (Table 1). The apparent specificity constant of *F. tularensis* MEP synthase is also comparable to the *Mycobacterium* and *Synechocystis* enzymes, although it is 20 fold less than that reported for the *E. coli* enzyme (due to the difference in *k_cat_^DXP^*). Assays performed with various divalent cations revealed that recombinant *F. tularensis* MEP synthase prefers MgCl_2_, although 60% of the enzyme activity is retained with MnCl_2_ (Fig. 5).

**Table 1:** MEP synthase Apparent Kinetic Parameters.

MEP synthase	*K_M_^DXP^* (µM)	*K_M_^NADPH^* (µM)	*k_cat_^DXP^* (s^−1^)	*k_cat_^NADPH^* (s^−1^)	*k_cat_^DXP^*/*K_M_^DXP^* (M^−1^min^−1^)	IC_50_^fosmidomycin^ (nM)	*K_i_* ^fosmidomycin^ (nM)	Refer.
*F. tularensis* a	103.7 +/− 12.1	13.3 +/− 1.5	2.0+/− 0.09	1.3+/− 0.04	1.2×10^6^+/− 9×10^4^	247	98.9 +/− 4.5	This study
*E. coli*	81–175	0.5–18	33	-	2.4×10^7^	35	21–215	[5], [33], [49], [50]
*M. tuberculosis*	47	29.7	1.2	-	1.5×10^6^	310	-	[51]
*Synechocystis* sp. PCC6803	170	3.5	17	-	6×10^6^	-	4	[16], [52]

a The values were calculated from data obtained in triplicate.

**Figure 6:**
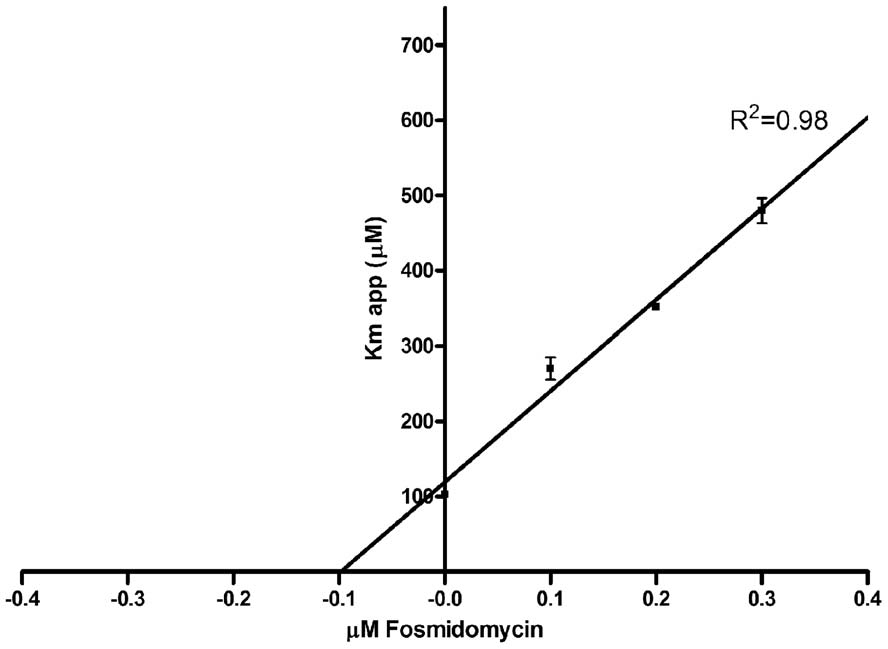
Graphical determination of the inhibition constant. Because fosmidomycin is a slow, tight binding inhibitor [5], the enzyme was preincubated with fosmidomycin for 10 minutes prior to addition of substrate. The absolute value of the x intercept of the line produced from linear regression fitting the plot of *K*_M_^app,DXP^ as a function of fosmidomycin concentration defined the *K_i_* as 98.9 +/− 4.5 nM. The R^2^ value is indicated.

Having established assay conditions for *F. tularensis* MEP synthase, we set out to evaluate the protein sensitivity to fosmidomycin. Half maximal activity (IC_50_) was observed at 247 nM, similar to that reported for the *Mycobacterium* homolog (Table 1). The *K_i_* for fosmidomycin (99 nM), obtained from a plot of *K_M_^app, DXP^* as a function of inhibitor concentration (Fig. 6), is greater than that reported for *E. coli* and *Synechocystis* sp. PCC6803 (Table 1). This may reflect structural differences in the MEP synthase homologs, although this remains to be determined.

### *F. tularensis* MEP synthase in a high-throughput assay

One method of identifying lead molecules in the drug development process involves the screening of large molecular libraries for inhibitors of an assay. A crucial issue for reliable high-throughput screening is the quality and robustness of the assay, often described in terms of the Z-factor [6]. An assay with a Z-factor score between 0.5 and 1.0 is considered excellent for high-throughput screening. To determine the Z-factor for the assay using *F. tularensis* MEP synthase, we adjusted the assay volume to accommodate a 96-well plate, fixed the substrate concentration at the *K_M_* (104 µM), used a saturating concentration of NADPH (150 µM), and evaluated three separate lots of purified MEP synthase in a series of assays performed over three consecutive days. Fosmidomycin was used as a positive control for inhibition. The Z-factor with *F. tularensis* MEP synthase was found to be 0.8, indicative of an assay (and enzyme) well suited for use in a high-throughput screen.

### Phosphorylation of *F. tularensis* MEP synthase

The first committed and principle regulatory step in the MVA pathway is catalyzed by HMG-CoA reductase (reviewed in [17]). Multifaceted control mechanisms regulate HMG-CoA reductase activity, including the modulation of enzyme concentration, the modulation of membrane composition/fluidity, and the regulation of enzyme activity via reversible phosphorylation (specifically, enzyme inhibition by phosphorylation of a serine residue in the catalytic domain [18]). In comparison to the MVA pathway, much less is known about the regulatory mechanisms that control metabolic flux through the MEP pathway. Engineered alterations of MEP pathway gene expression suggest that several MEP pathway enzymes may share control over metabolic flux (reviewed in [19]). The role of posttranslational modification, such as reversible phosphorylation, remains unknown. Thus, we sought to evaluate if a phosphorylation site on *F. tularensis* MEP synthase could be identified.

**Figure 7:**
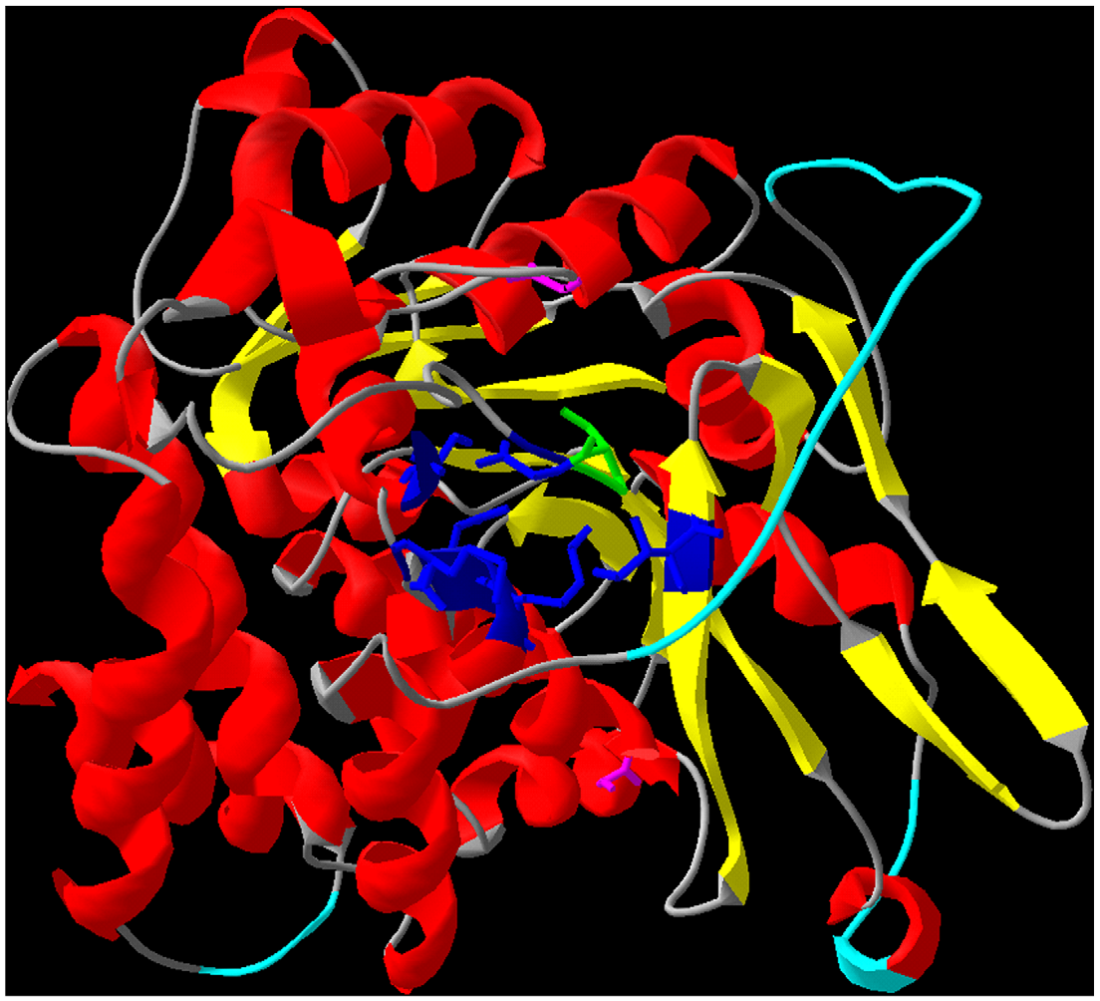
Predicted tertiary structure of *F. tularensis* MEP synthase, homology-modeled using SWISS-MODEL. A crystal structure of *F. tularensis* MEP synthase has not been reported. To permit the visualization of phosphoserine177 within the context of the tertiary structure, the *F. tularensis* MEP synthase was modeled based upon the resolved structure of the *E. coli* homolog[48] (48% identity, 66% homology). A cartoon representation of the model is shown, with alpha helices colored red, beta sheets colored yellow, and coiled regions colored gray. The beta sheets comprise the dimer interface in the *E. coli* structure. The quality of the model was evaluated with ProQRes (Fig. S3) which provides scores ranging from 0 (unreliable) to 1 (reliable). Regions of the model scoring <0.5 are colored light blue. Primary sequence alignment and the structure of *E. coli* MEP synthase were used to identify residues comprising the substrate binding site (colored dark blue with backbone and sidechain residues shown). Serine177 (colored green with backbone and sidechain residues shown) is equivalent to *E. coli* Ser186, which contributes to the substrate binding site and has been shown to participate in conformational changes that occur upon substrate binding [13]. Two tryptophan residues present in the *F. tularensis* MEP synthase model are colored pink.

**Figure 8:**
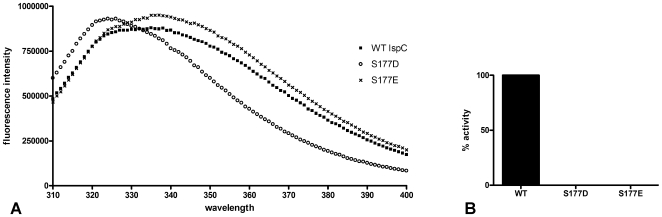
Regulation of *F. tularensis* MEP synthase. A) Intrinsic fluorescence spectra of MEP synthase and its mutants. Wildtype and mutant (S177D and S177E) proteins were adjusted to 5 µM in 0.1 M Tris pH 7.5, 1 mM NaCl, 5 mM DTT and analyzed using an excitation wavelength of 290 nm. The emission spectra was measured from 310 to 400 nm. The Em λ_max_ of wildtype MEP synthase was detected at 335 nm, of S177E was detected at 337 nm, and of S177D was detected at 326 nm. The blue shift observed with S177D is indicative of a conformational change sequestering tryptophan residues into a hydrophobic environment. The slight red shift observed with S177E is indicative of a conformational change exposing tryptophan residues to a hydrophilic environment. The increased quantum yield observed with both S177D and S177E is also indicative of a structural change in MEP synthase. B) The relative catalytic activity of wildtype MEP synthase and the S177D and S177E mutants. Assays were performed with 300 µM DXP and 150 µM NADPH.

Recombinant *F. tularensis* MEP synthase was purified from *E. coli* (induced with 10 µM IPTG), subjected to trypsinization, and phosphopeptides were isolated and identified via titanium dioxide chromatography-tandem mass spectrometry. Phosphoserine 177 (equivalent to Ser186 in the *E. coli* enzyme) was identified (Fig. 7). *E. coli* Ser186 is located in the substrate binding site, directly interacts with the substrate, and participates in additional interactions that contribute towards protein conformational changes that occur upon substrate binding [13]. Serine 177 presumably has a similar role in the *F. tularensis* homolog. Phosphorylation of Ser177 is likely to disrupt these interactions, influencing substrate binding and protein conformation, ultimately affecting enzyme activity. To test this assumption, two mutants of *F. tularensis* MEP synthase were created, S177D and S177E, wherein Ser177 was changed to an aspartate or glutamate, respectively, which serve to mimic a phosphoserine. Each mutant was expressed in *E. coli* and purified to near homogeneity via a C-terminal His-tag. Relative to wildtype MEP synthase, a blue shift in the intrinsic fluorescence maximum of S177D suggests changes in the protein globular fold are brought about by the introduction of the Asp residue (Fig. 8A). The slight red shift in fluorescence emission spectrum of S177E is also indicative of a conformational change, although the change in S177E suggests exposure of a tryptophan residue to a hydrophilic environment whereas the blue shift observed with S177D is indicative of a conformational change sequestering a tryptophan in a hydrophobic environment. Like the wildtype enzyme, both mutants exist as dimers in solution, as determined by size-exclusion chromatography (Fig. S2). Enzyme assays performed using the purified mutants reveal that each is inactive (Fig. 8B). Collectively, these results suggest that phosphorylation of Ser177 leads to a conformational change in *F. tularensis* MEP synthase which inhibits the enzyme and may serve as a control mechanism to regulate metabolic flux through the MEP pathway.

### Conclusions

In conclusion, we have shown that *F. tularensis* MEP synthase is a valid target for the development of novel therapeutics. Inhibition of MEP synthase is sufficient to inhibit the growth of *F. tularensis in vitro*. Purified MEP synthase is kinetically active and readily lends itself to use in high-throughput screens. Furthermore, our investigation is the first to show that metabolic flux through the *F. tularensis* MEP pathway may be regulated by phosphorylation of MEP synthase, similar to the regulatory control observed in the MVA pathway.

## Supporting Information

Figure S1 Growth curves of *F. tularensis* subsp. *novicida* in minimal media supplemented with the indicated concentration of fosmidomycin (FoS). All conditions were evaluated in duplicate.
(3.20 MB TIF)

Figure S2 Molecular weight determination by size-exclusion chromatography. Protein standards (▪) were used to calibrate the column. Linear regression fitting (R^2^ is indicated) generated the standard curve, which was used to determine the molecular weight of IspC, S177D, and S177E.
(1.44 MB TIF)

Figure S3 ProQRes evaluation of the *F. tularensis* MEP synthase structural model generated by SWISS-MODEL. ProQRes uses atom-atom contacts, residue-residue contacts, solvent accessibility, and secondary structure information to score the model over a sliding window of 9 residues[10]. Scores range from 0 (unreliable) to 1 (reliable).
(3.95 MB TIF)
