# Supplementary figures and images for: Kinetic Characterization and Phosphoregulation of the Francisella tularensis 1-Deoxy-D-Xylulose 5-Phosphate Reductoisomerase (MEP Synthase)
Source: PLoS One. 2009 Dec 14;4(12):e8288. doi: 10.1371/journal.pone.0008288 (PMC2788227; doi:10.1371/journal.pone.0008288)

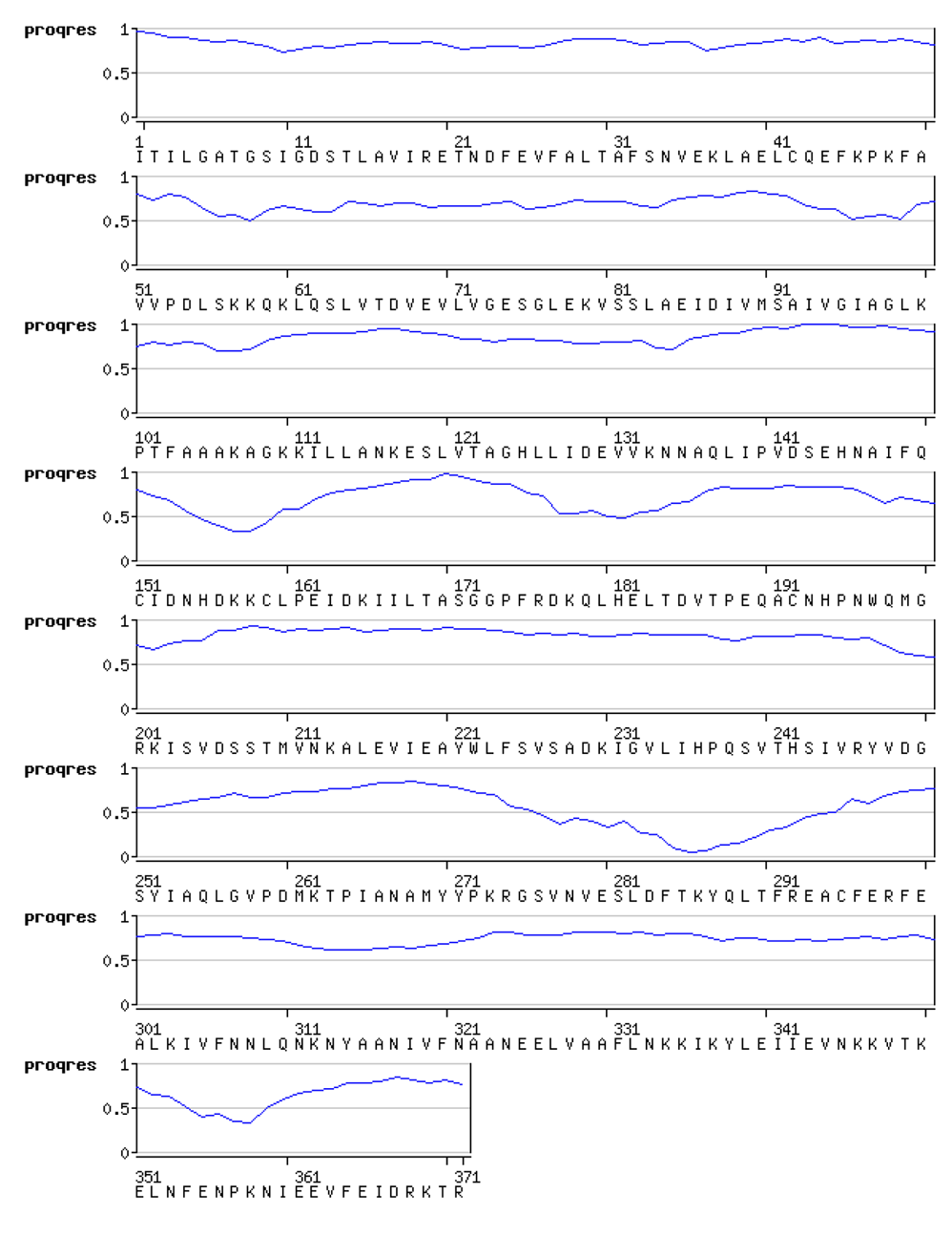

Supplement: Figure S3 — ProQRes evaluation of the F. tularensis MEP synthase structural model generated by SWISS-MODEL. ProQRes uses atom-atom contacts, residue-residue contacts, solvent accessibility, and secondary structure information to score the model over a sliding window of 9 residues[10]. Scores range from 0 (unreliable) to 1 (reliable). (3.95 MB TIF) [file pone.0008288.s003.tif]
